# Transcriptional ontogeny of the developing liver

**DOI:** 10.1186/1471-2164-13-33

**Published:** 2012-01-19

**Authors:** Janice S Lee, William O Ward, Geremy Knapp, Hongzu Ren, Beena Vallanat, Barbara Abbott, Karen Ho, Seth J Karp, J Christopher Corton

**Affiliations:** 1 National Health and Environmental Effects Research Laboratory, United States Environmental Protection Agency, Research Triangle Park, NC 27711, USA; 2 National Center for Environmental Assessment, United States Environmental Protection Agency, Research Triangle Park, NC 27711, USA; 3 Department of Medicine, Genomics Core, Beth Israel Deaconess Medical Center, Boston, Massachusetts 02215, USA

## Abstract

**Background:**

During embryogenesis the liver is derived from endodermal cells lining the digestive tract. These endodermal progenitor cells contribute to forming the parenchyma of a number of organs including the liver and pancreas. Early in organogenesis the fetal liver is populated by hematopoietic stem cells, the source for a number of blood cells including nucleated erythrocytes. A comprehensive analysis of the transcriptional changes that occur during the early stages of development to adulthood in the liver was carried out.

**Results:**

We characterized gene expression changes in the developing mouse liver at gestational days (GD) 11.5, 12.5, 13.5, 14.5, 16.5, and 19 and in the neonate (postnatal day (PND) 7 and 32) compared to that in the adult liver (PND67) using full-genome microarrays. The fetal liver, and to a lesser extent the neonatal liver, exhibited dramatic differences in gene expression compared to adults. Canonical pathway analysis of the fetal liver signature demonstrated increases in functions important in cell replication and DNA fidelity whereas most metabolic pathways of intermediary metabolism were under expressed. Comparison of the dataset to a number of previously published microarray datasets revealed 1) a striking similarity between the fetal liver and that of the pancreas in both mice and humans, 2) a nucleated erythrocyte signature in the fetus and 3) under expression of most xenobiotic metabolism genes throughout development, with the exception of a number of transporters associated with either hematopoietic cells or cell proliferation in hepatocytes.

**Conclusions:**

Overall, these findings reveal the complexity of gene expression changes during liver development and maturation, and provide a foundation to predict responses to chemical and drug exposure as a function of early life-stages.

## Background

The liver is the largest internal organ and provides many essential metabolic, exocrine and endocrine functions. The use of animal models including the mouse and primary cell cultures has identified many of the genes and pathways regulating embryonic liver development. These studies show that much of hepatogenesis is conserved throughout evolution. The liver, as well as the pancreas, develops from two unique spatial domains of the definitive endodermal epithelium of the embryonic foregut. Fate-mapping experiments have shown that the liver arises from lateral domains of endoderm in the developing ventral foregut as well as from endodermal cells that track along the ventral midline [1,2]. During closure of the foregut, the medial and lateral domains come together as the hepatic endoderm is specified. The pancreas is also induced in lateral endodermal domains, adjacent and caudal to the lateral liver domains, as well as in cells near the dorsal midline of the foregut [3,4]. After the domains are specified and initiate morphogenetic budding, the dorsal and ventral pancreatic buds merge to create the gland. These events occur at 8.5 days of mouse gestation (GD8.5), corresponding to about 3 weeks of human gestation. Despite differences in how the different progenitor domains are specified, descendants of both pancreatic progenitor domains make endocrine and exocrine cells, and descendants of both liver progenitor domains contribute to differentiating liver bud cells [1,2].

Newly specified hepatic cells in embryos are referred to as hepatoblasts which express albumin (*Alb*), transthyretin (*Ttr*) and α-fetoprotein (*Afp*) at about the 7 somite (7S) stage of mouse development (approximately GD8.25). Hepatoblasts are bipotential; those residing next to portal veins become bile epithelial cells that will line the lumen of the intrahepatic bile ducts while most of the hepatoblasts in the parenchyma differentiate into hepatocytes. The maturation of functional hepatocytes and the formation of a biliary network connected to the extrahepatic bile ducts are gradual, beginning at GD13 and continuing until after birth [2].

Between GD9.5 and GD15 the liver bud undergoes substantial growth and becomes the major site of fetal hematopoiesis. Erythrocytes are required for survival and growth of the mammalian embryo beyond early post-implantation stages of development. The embryo's first "primitive" erythroid cells, derived from a transient wave of committed progenitors, emerge from the yolk sac as immature precursors and differentiate as a semisynchronous cohort in the bloodstream [5]. The yolk sac also synthesizes a second transient wave of "definitive" erythroid progenitors that enter the bloodstream and seed the fetal liver. Simultaneously, hematopoietic stem cells within the embryo also seed the liver and are the presumed source of long-term erythroid potential. Fetal-definitive erythroid precursors mature in macrophage islands within the liver, enucleate, and enter the bloodstream as erythrocytes. Toward the end of gestation, definitive erythropoiesis shifts to its final location, the bone marrow [6].

Fetuses and neonates are generally considered more susceptible to xenobiotics than adults [7]. Pharmacokinetic differences in the fetus, newborns and children may alter responses to environmental chemicals compared to adults, potentially resulting in a different spectrum of susceptibility to adverse health effects. Detoxification and elimination of xenobiotics is a major function of the liver and is important in maintaining the metabolic homeostasis of the organism. Xenobiotics are metabolized by a large number of xenobiotic metabolizing enzymes and transporters which fall into three broad categories: phase I, phase II and transporters. Phase I enzymes are involved in oxidation, reduction, and hydrolysis, and include cytochrome P450 family members. Phase II enzymes convert the products of phase I metabolism into amphiphilic anionic conjugates that are water soluble and include glutathione transferases, UDP-glucuronyl transferases, and sulfotransferases. Phase III transporters export conjugated xenobiotics out of the liver and include ATP binding cassette subfamily members, organic anion and cation transporters, and solute carriers [8]. A large number of genetic and biochemical studies have shown that the level of expression and activity of individual XMEs in part determines the fate of a specific xenobiotic and whether exposure results in toxicity [9,10]. A comprehensive understanding of the differences between fetal and neonatal XME gene expression with that in the adult would be useful to predict classes of chemicals to which these life stages may exhibit altered responses.

Liver gene expression during development has been examined previously. Jochheim et al. [11] studied gene expression profiles in GD7.5, GD11.5, and GD13.5 BALB/C mice using microarrays of ~12,000 genes. The greatest number of differentially regulated genes (3063) was found in GD11.5 versus adult liver, and the lowest number was found in GD11.5 versus GD13.5 (517) [11]. Gene expression changes were observed for a number of genes known to be involved in liver development, including *Afp*, *Alb*, *C/EBP alpha*, *C/EBP beta*, *GATA-4 *and *Hex*. Jochheim-Richter et al. [12] subsequently performed a cluster analysis of livers from GD9.5, GD11.5, and GD13.5 BALB/C mice. One hundred and thirty genes were continuously expressed at all stages of development with peak expression of 44 genes at GD9.5. Li et al. [13] studied gene expression and transcriptional regulation at 14 time points across the C57/B6 mouse liver development, from GD11.5 to adult. Cell-cycle-related genes were highly expressed in early embryo development, defense-related genes were activated around birth, and liver-function-related transcription factors and genes were highly activated in the later stage of development [13].

Using full-genome arrays, we determined the transcriptional ontogeny of the developing liver from GD11.5 to the adult. We examined the expression of genes important in liver differentiation, hematopoiesis, and xenobiotic metabolism. Although liver gene expression during development has been examined previously, as described above, we employed a number of strategies to provide insights into the function of the genes identified including comparing the liver profiles to the profiles generated from at least 100 different mouse tissues and to those profiles that were derived from different types of blood cells. In addition, we describe in greater detail the relationships between the changes in those genes involved in chemical metabolism and transport and possible phenotypic effects of chemical exposure.

## Methods

### Animals and study design

#### Study 1- samples for microarray

In Study 1, timed-pregnant C57BL/6J dams were purchased from Charles River Laboratory (Raleigh, NC) and acclimated for 1 week. Mice were housed (1 per cage) in polycarbonate cages on Alpha-dri bedding (Shepherd Specialty Papers, Kalamazoo, MI). Animal facilities were controlled for temperature (20-24°C) and relative humidity (40-60%), and kept under a 12-h light-dark cycle. The basal diet was pellet chow (LabDiet 5001, PMI Nutrition International LLC, Brentwood, MO) and tap water was provided ad libitum. Pregnant dams were sacrificed at gestational day (GD) 19 and male pups were sacrificed by decapitation. Mice from additional litters at ages PND7, PND30, and PND67 were sacrificed using CO_2 _asphyxiation. Only male mice were used in this study. Livers were removed, weighed and sections from the left and median lobes were fixed in formalin, embedded, sectioned, and stained with H&E. The remainder of the liver was cubed and stored at -80°C until RNA isolation.

#### Study 2- samples for Real-Time RT-PCR

RNA samples used for real-time RT-PCR were obtained from another study run by a collaborating author. In Study 2, timed pregnant CD-1 mice were obtained from Charles River Laboratories (Raleigh, NC), where females were bred overnight, and the sperm positive females were designated GD0. Pregnant mice were shipped to EPA on GD0 and upon arrival were housed individually in polypropylene cages with Alpha-dri (Shepherd Specialty Papers, Kalamazoo, MI) bedding and provided pellet chow (LabDiet 5001, PMI Nutrition International LLC, Brentwood, MO) and tap water *ad libitum*. Animal facility conditions were the same as in Study 1. Pregnant mice were killed on GD14 and GD17 for collection of adult and fetal livers. Fetal livers were pooled by litter because these tissues were small and determination of sex of the fetuses was not feasible, as it would have required extensive additional work. Remaining dams were allowed to deliver pups and tissues were collected from pups on PND 1, 7, 14, 21, and 28. Since fetal livers were pooled, with male and female expected to be represented equally for each litter, a decision was made to also pool the postnatal livers with one male and one female per litter combined for RNA preparation. All litters were weaned on postnatal day (PND) 21 at which time liver was also collected from the dams. At collection, livers were frozen in RNA-Later (Ambion Inc, Austin, TX) at -80°C until RNA isolation.

All animal studies were conducted in accordance with guidelines established by the U.S. EPA ORD/NHEERL Institutional Animal Care and Use Committee. Procedures and facilities were consistent with the recommendations of the 1996 NRC "Guide for the Care and Use of Laboratory Animals", the Animal Welfare Act, and Public Health Service Policy on the Humane Care and Use of Laboratory Animals.

### RNA isolation

Total RNA was isolated from mouse livers using TRI reagent (Sigma Chemical, St. Louis, MO) according to the manufacturer's directions, and further purified using the Qiagen RNeasy mini RNA cleanup protocol (Qiagen, Valencia, CA). RNA pellets were stored in 70% ethanol at -80°C until further use. The integrity of each RNA sample was determined using an Agilent 2100 Bioanalyzer (Agilent, Foster City, CA), and RNA quantity was determined using a Nanodrop^® ^ND-100 (NanoDrop Technologies, Wilmington, DE).

### Microarray hybridizations

Liver gene expression analysis was performed according to the Affymetrix recommended protocol using Affymetrix Mouse Genome 430 2.0 GeneChips^® ^containing probes for over 30,000 well-characterized genes. Total RNA (5 μg per sample) was labeled using the Affymetrix^® ^One-Cycle cDNA Synthesis protocol and hybridized to arrays as described by the manufacturer (Affymetrix^®^, Santa Clara, CA). Microarray hybridizations were conducted overnight at 45°C while rotating in an Affymetrix hybridization oven. After 16 hours of hybridization, the cocktail was removed and the arrays were washed and stained in an Affymetrix GeneChip^® ^fluidics station 450 according to the Affymetrix-recommended protocol. Arrays were scanned on an Affymetrix GeneChip^® ^scanner. Four mice per age group (from Study 1) were examined.

### Analyses of Microarray data

All Affymetrix (Santa Clara, CA) .cel files were first analyzed by Bioconductor's SimpleAffy package to assess data quality [14]. All .cel files passed this QC step. Data (.cel files) were background corrected and statistically filtered using Rosetta Resolver^® ^version 7.1 software (Rosetta Inpharmatics, Kirkland, WA). The background correction was done by Resolver's specific data processing pipeline (Affymetrix Rosetta-Intensity Profile Builder). Statistically significant genes were identified using one-way ANOVA with a false discovery rate (Benjamini-Hochberg test) of ≤ 0.05 followed by a post-hoc test (Scheffe) for significance. Hierarchical clustering was performed using CLUSTER and visualized with TREEVIEW [15]. A detailed description of the microarray experiment is available through Gene Expression Omnibus at the National Center for Biotechnology Information at http://www.ncbi.nlm.nih.gov/geo/, as accession number GSE21224.

### Reanalysis of published microarray data

The raw data files analyzed in this project (.cel files from Affymetrix DNA chips) were downloaded from Gene Expression Omnibus (GEO). All of the .cel files were analyzed as described above. A detailed description of each experiment is available through Gene Expression Omnibus at the National Center for Biotechnology Information at http://www.ncbi.nlm.nih.gov/geo/.

To create a broader view of gene expression changes during development, we combined our dataset from the GD19 - PND67 male C57BL/6 mice with a dataset from CD-1 mice at GD11.5, GD12.5, GD13.5, GD14.5 and GD16.5 in which 10-week-old females were used as controls ([16]; GDS2577). Given differences in the experiments, we used Distance Weighted Discrimination (DWD) [17] to minimize systematic microarray data biases attributable to RNA source, different analysis laboratories, different microarrays, or other systematic differences which include sex and strain in this case. DWD applies Singular Value Decomposition amended with Fisher Linear Discrimination to find better corrections for systematic effect adjustments. These adjustments were applied to preserve variation not caused by systematic effects. Based on evaluation of Affymetrix spike-in controls, the samples from the two experiments exhibited similar behavior by PCA after the procedures were applied (Additional File 1). The resulting merged dataset was used for global analysis of fetal and neonatal expression as well as the analysis of pancreas-specific genes, hematopoietic-specific genes and xenobiotic metabolism genes. In this merged dataset, the fetal and neonatal samples were normalized to the adult samples from the two studies. The data was also analyzed by TightCluster, a resampling-based approach for identifying stable and tight patterns in data [18]. We used only the GD19 - PND67 dataset generated from male C57BL/6J mice (Study 1) to perform the analysis of the time course of pancreas-specific genes. In this case the DWD was not applied, but rather we used the Rosetta Resolver procedures as detailed above. The fetal (GD19) and neonatal (PND7, PND30) samples were compared to the PND67 adult samples.

Genes were divided into those that were up- or down-regulated and were analyzed separately for enrichment of canonical and toxicity pathways using Ingenuity Pathway Analysis (IPA). Pathways that did not meet the p-value significance (p ≥ 0.05) were excluded. P-values were converted to -Log(p-value). For those significant pathways derived from down-regulated genes all -Log(p-value)s were converted to negative numbers. All data was clustered and visualized as described above using Cluster and TreeView.

The microarray data from the liver samples were compared to a database of > 80 other mouse tissues (GNF Mouse GeneAtlas V3; GEO ID GSE10246). Comparisons were made in Rosetta Resolver using PCA of all tissues and hierarchical clustering comparing the liver and pancreas samples. Tissue-specific signature genes were generated from this dataset by identifying those genes which exhibited differential expression between either 1) the top 1000 adult pancreas genes (p < 3.16E-05), 2) the top 500 fetal liver genes (p < 2.55E-08), or 3) the top 500 bone marrow genes (p < 3.07E-04) and all other tissues in the dataset.

Human samples were derived from the following ArrayExpress submissions: E-TABM-185, E-AFMX-5, E-MTAB-24, and E-MTAB-25. They included 3 fetal livers, 10 adult livers and 5 adult pancreases. Hierarchical clustering was performed in Rosetta Resolver. Mouse-human comparisons of canonical pathways were made using IPA as described above. Genes were divided into those 1) expressed in fetal liver only, 2) common to pancreas and fetal liver with the fetal liver fold-change values and 3) in pancreas only. These 3 groups were analyzed using IPA before and after separation into up- and down-regulated genes. The same canonical pathways were compared between mice and humans.

We examined the expression of genes identified as signature genes for purified erythropoeitic cells including natural killer (NK) cells, T cells, B cells, monocytes, neutrophils, nucleated erythrocytes, and activated and naive CD4+ (helper) and CD8+ (cytotoxic) T cell subsets ([19]; GSE6506). The signature genes were derived from the Chambers et al. [16] study.

### Evaluation of selected genes by Real-Time RT-PCR

Expression of 10 genes (*A2m, Afp, Hamp2, Reep5, Slc39a5, Spink3, Alas2, Epor, Gata1, Klf1*) was confirmed using the tissues from Study 2 by quantitative RT-PCR, validating our microarray analysis procedures. Quantitative RT-PCR was performed on RNA from the eight age groups (2 prenatal, 5 postnatal, and 1 adult age group with four samples per group) in Study 2 (described above). Eighty-five nanograms of total RNA were loaded into each one-step qRT-PCR reaction containing 1X QuantiTect™ Probe RT-PCR master mix (Qiagen, Valencia, CA) and 1 × TaqMan^® ^Gene Expression Assays (Applied Biosystems, Foster City, CA) for the desired gene target. We examined the expression of two housekeeping genes (*Actb, Gapdh*) throughout development and showed that expression changed significantly with greatest expression for both genes between GD14 and GD17, indicating that these genes were not appropriate normalization controls (Additional File 2). Therefore, a relative standard curve was generated for each target using serially diluted RNA pooled from the study. Relative quantities were calculated from the appropriate relative standard curve. Relative quantities were then compared among treatment groups. Statistical significance was determined using Tukey-Kramer HSD.

### In situ hybridization

Embryos were fixed in 4% paraformaldehyde and embedded in paraffin. Sections were cut, deparaffinized, treated with proteinase K (10 ug/mL) for 10 minutes, post-fixed with 4% paraformaldehyde and acetylated with triethanolamine buffer (pH 8.0) containing 0.25% acetic anhydride for 10 minutes. After rinsing, slides were hybridized with antisense and sense DIG-labeled RNA probes. Probes were 600-700bp PCR products amplified from embryonic liver cDNA libraries using gene specific primers. Gel-purified PCR products were subcloned and labeled using a DIG RNA-labeling mix (Roche Applied Science, Indianapolis, IN). Slides were hybridized with labeled sense and anti-sense probes in 50% formamide, 10% dextran sulfate, 1x Denhardt's solution, 200ug/mL tRNA, 10mM Tris pH 7.5, 600mM NaCl, 1mM EDTA, 0.25% SDS in a humidified chamber overnight at 65 degrees C. Slides were washed in 5X SSC, 2X SSC and 0.2X SSC at 50 degrees C. DIG was detected with an alkaline-phosphatase-conjugated antibody (Roche Applied Sciences) and nitroblue tetrazolium chloride-5-bromo-4-chloro-3-indoylphosphate toludidine salt.

## Results and Discussion

### Transcriptional ontogeny of the developing mouse liver

**Figure 1:**
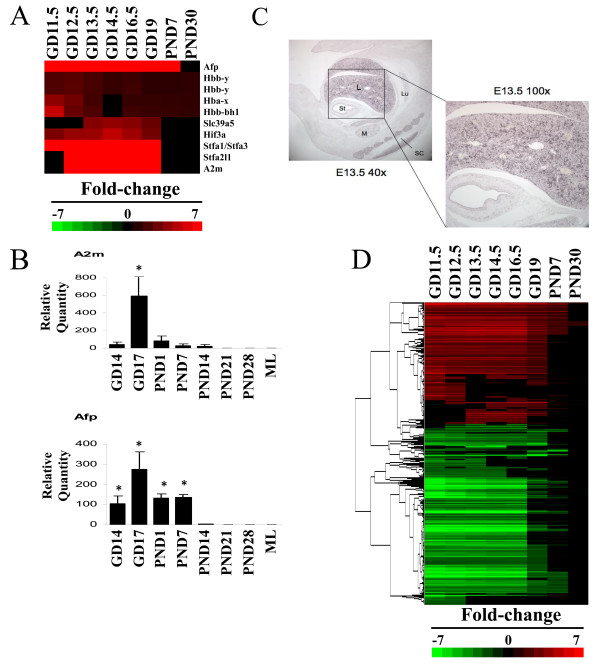
**Transcriptional ontogeny of the developing mouse liver**. A. Expression changes in fetal liver genes. Fetal liver genes were identified as detailed in the Materials and Methods. The intensity scale indicates fold-changes compared to the adult controls. Red, up-regulation; green, down-regulation; black, no change. B. RT-PCR of fetal liver gene expression in mouse livers from GD14 to PND28. *, indicates statistical significance of expression changes relative to PND28 (p ≤ 0.05); ML, maternal liver. C. In situ hybridization of *Makorin 1 *(*Mkrn1) *in the GD13.5 fetus. L, liver; Lu, lung; St, stomach; M, metanephros; SC, spinal cord. D. Global gene expression in the developing mouse liver. Genes which exhibited significant differences in expression compared to adult animals were identified as detailed in the Materials and Methods.

Gene expression was measured in the livers from fetuses (gestation day (GD) 11.5-19) and neonates (postnatal day (PND) 7 and 30) and compared to that in adult livers. Marker genes for the fetal liver were examined for predicted expression behavior. Fetal liver-specific genes which exhibited significant differences (p < 2.55E-08) between fetal liver and ~80 other mouse tissues were initially examined for changes. Figure 1A shows a subset of the genes which exhibited increased expression in the fetal liver compared to the adult. The genes included alpha fetoprotein (*Afp*), widely recognized as a fetal liver protein [20], a number of fetal hemoglobin genes (*hemoglobin Y, beta-like embryonic chain; hemoglobin X, alpha-like embryonic chain in Hba complex; hemoglobin Z, beta-like embryonic chain*) and alpha-2-macroglobulin (*A2m*), known to be expressed in the fetal liver [21]. Genes not previously associated with fetal expression were also identified including stefin family members (*stefin A1/A3 *and *stefin A2 like 1*) containing cysteine protease inhibitor domains, *hypoxia inducible factor 3, alpha subunit *(*Hif3a*) and the *zinc transporter solute carrier family 39 (metal ion transporter), member 5 (Slc39a5)*. The fetal expression of *Afp *and *A2m *(Figure 1B) and *Slc39a5 *(discussed below) was confirmed by RT-PCR using an independent set of livers from fetal and neonatal mice. Genes that were down-regulated specifically in the fetal liver were also identified (Additional File 3). Expression of the *E3 ubiquitin ligase Makorin *(*Mkrn1*) and a regulator of splicing, *Regulator of differentiation 1 *(*Rod1*) was examined by in situ hybridization (ISH). By microarray, both genes exhibited maximal expression at GD14.5 (Additional File 4, Figure S1). Expression of the genes in the liver by ISH could be detected as early as GD10.5 through GD15.5 (Figure 1C and Additional File 4, Figures S2-S6). Thus, the microarray results were consistent with the analysis of RNA expression by other methods.

**Figure 2:**
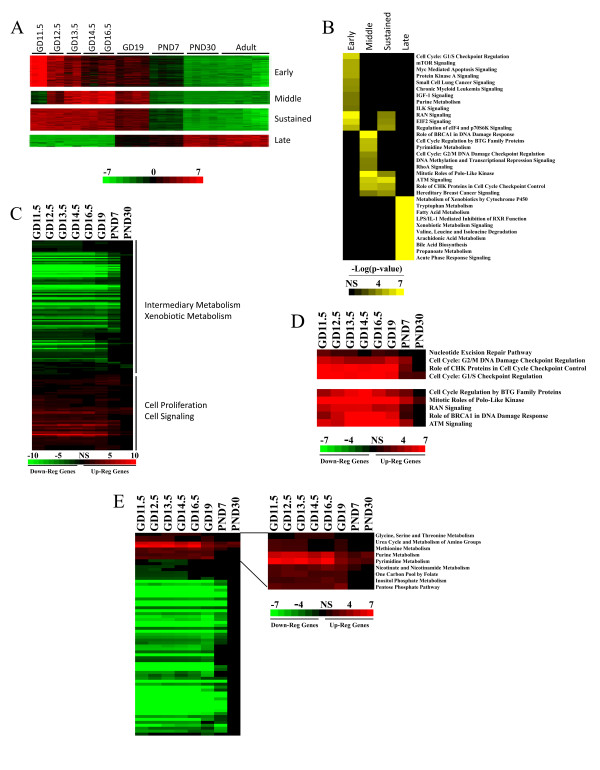
**Canonical pathways altered in the developing liver**. A. TightCluster groups genes into 4 temporal categories during liver development. Shown are examples of clusters that group genes into one of the 4 temporal categories. B. Genes expressed at different times during development fall into unique canonical pathways. Genes in the 4 temporal groups identified using TightCluster (Figure 2A) were analyzed using IPA. Only the top 10 significant pathways in the late group are shown. C. Global view of canonical pathways altered during development. Canonical pathways significantly altered at the indicated times during development compared to adult mice were identified using IPA. D. Increased expression of genes in canonical pathways involved in DNA maintenance and cell cycle (top) and cell fate signaling (bottom). E. Changes in canonical pathways of intermediary metabolism. Left, all significantly altered pathways of metabolism. Right, up-regulated pathways. For B-E, the scale numbers are the -log(p-value) and range from < 10^-10 ^to not significant (NS). Yellow, altered pathway using all genes as input; red, up-regulated pathway using all up-regulated genes as input; green, down-regulated pathway using all down-regulated genes as input; black, not significant.

Transcriptional ontogeny of the developing liver was characterized by examining the expression of all genes that exhibited changes during development (Figure 1D and Additional File 5). Approximately 4370 genes exhibited altered expression in at least one of the time points. A greater number of genes were under-expressed than over-expressed relative to the adults. The gene expression differences were most striking between GD11.5-16.5. Petkov et al. [22] also found the gene expression of fetal hepatoblasts to differ profoundly from that of adult hepatocytes, with a major switch at GD16 to 17. GD19, PND7 and PND30 time points exhibited progressively fewer genes and in general, smaller fold-change differences compared to the adult animals. While many genes exhibited striking differences throughout all or most periods of development, groups of genes were identified that were altered only during discrete windows of development (Figure 2A). Four major groups were identified including 639 genes whose maximal expression was between GD11.5 and GD12.5 (early expression), 851 genes whose maximal expression was between GD14.5 and GD16.5 (middle expression), 236 genes which exhibited sustained and approximately uniform expression throughout development (GD11.5-GD16.5; sustained expression) and 1423 genes which exhibited maximal expression in the adult (late expression).

Genes expressed early in development included those expressed in embryonic stem (ES) cells and are involved in tissue development including *Mdk, Ptn, Hmga2, Ndn*, and *Pa2g4*. Midkine (*Mdk*) and the related cytokine pleiotrophin/heparin-binding growth-associated molecule (*Ptn*) are essential for normal development of the catecholamine and renin-angiotensin pathways. *Mdk *regulates *Ptn *expression [23], and *Ptn *may be secreted from embryonic mesenchymal cells as a mitogen of parenchymal cells in the embryonic liver [24]. The high mobility group AT-hook 2 gene (*Hmga2*), abundant in ES cells is involved in transcriptional activation of cell proliferation genes, substantially contributing to the plasticity of ES cell chromatin and maintenance of an undifferentiated cell state [25]. Necdin (*Ndn*) preferentially expressed in primitive stem cells, is an important protein in hematopoietic stem cell regulation [26]. Proliferation-associated 2G4 (*Pa2g4*) is expressed in mouse ES cells [27]. The entire list of genes which exhibited expression during discrete windows of development is found in Additional File 6.

To categorize the pathways altered during liver development, Ingenuity Pathways Analysis (IPA) was used to identify the canonical pathways that were significantly altered during development. In the first analysis, the four sets of genes which exhibited discrete windows of expression were examined (Figure 2B). The canonical pathways that were significantly altered were, for the most part, unique for the early, middle or late time periods of development. Pathways altered late in the adult were those associated with functions of the mature liver including intermediary metabolism, whereas almost all of the pathways altered in the fetus were associated with signaling pathways that control cell proliferation, active during the growth of the liver bud.

To assess the impact of liver development on putative pathway activation or suppression, IPA was again used to identify pathways significantly altered at each time point in development using those genes described in Figure 1D. The genes at each time point were separated into those that were up- or down-regulated relative to the adult and were analyzed separately as detailed in the Materials and Methods. Pathways regulated in the fetus were dominated by those that were less active than in the adult including those involved in intermediary and xenobiotic metabolism, whereas putative activated pathways were dominated by those involved in cell proliferation and cell signaling (Figure 2C). Activated pathways were associated with DNA replication fidelity during the cell cycle (G1/S checkpoint regulation, cell cycle G2/M DNA damage checkpoint regulation, role of CHK proteins in cell cycle checkpoint control, nucleotide excision repair pathway) (Figure 2D, top). Li et al. [13] also found that in early embryo development, cell-cycle-related genes were highly expressed and defense-related genes were activated around birth. Pathways with well-known effects on cell proliferation and apoptosis were significantly regulated including those under control of 14-3-3 regulatory proteins, p53, polo-like kinase, BTG family members, BRCA1, ATM, and OCT4 (Figure 2D, bottom and Additional File 7). A number of liver toxicity pathways were also significantly modified including increases in liver hematopoiesis (discussed below) and hemorrhaging and down-regulation of liver cholestasis associated with bile acid homeostasis (Additional File 8). An examination of pathways involved in xenobiotic and intermediary metabolism (Figure 2E) showed that while most pathways were suppressed during development, nine were up-regulated, the most prominent of those being purine and pyrimidine metabolism, likely activated to support DNA and RNA synthesis during active liver growth (Additional File 9, Figures S7 and S8).

This analysis highlights the dramatic changes the fetal liver undergoes during development. We identified key pathways that support the growth and function of the developing liver. These gene expression and pathway changes will be a useful resource for hypothesis generation and testing of the role of genes, pathways and genetic networks in liver development.

### Transcriptional similarities between the developing liver and the pancreas

**Figure 3:**
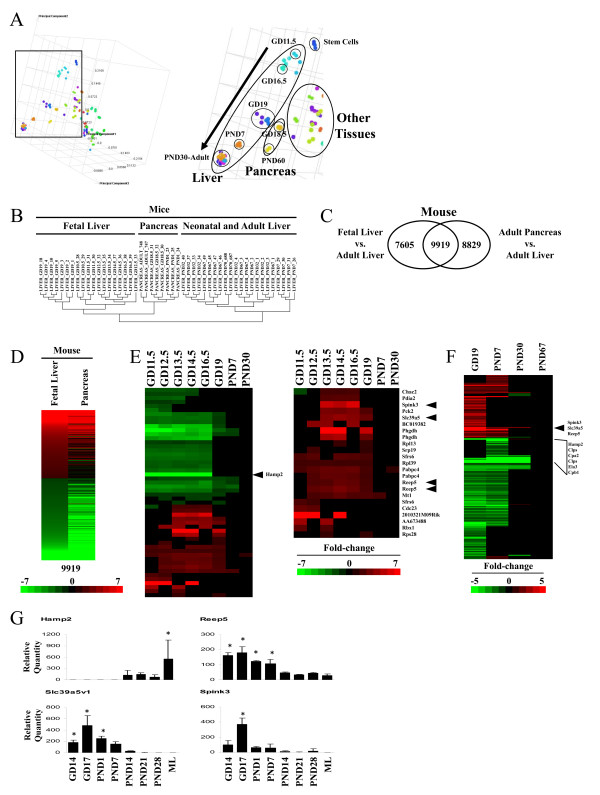
**Transcriptional similarities between the fetal liver and pancreas in the mouse**. A. Principal component analysis (PCA) of fetal and neonatal liver compared to a library of ~80 mouse tissues. Left, view of all mouse tissues used in the comparison. Right, enhanced view showing the trajectory of liver maturation (arrow) from stem cells to adult livers and similarity between GD19 livers and pancreas from GD18.5 and adult animals. B. Fetal liver exhibits greater similarity to pancreas than adult liver. The biological replicates were clustered using hierarchical clustering. C. Overlap in the genes differentially expressed in the fetal liver or pancreas compared to the adult liver. Fetal liver (GD19) or adult pancreas (PND60) was compared to adult livers. D.Concordance in the direction and intensity of the fold-changes in the 9919 overlapping genes from C. E. Expression of pancreas-specific genes in the developing liver. Left, expression of all genes identified as detailed in the Materials and Methods. The position of *Hamp2*, examined by RT-PCR is shown. Right, pancreas-specific genes up-regulated during development. Arrowheads indicate genes examined by RT-PCR. F. Sustained expression of a subset of pancreas-specific genes in the neonate. The expression of the pancreas-specific genes was examined in the C57BL/6J male mice at the indicated times in the fetus and neonate. For D-F, the intensity scale indicates fold-changes compared to the adult controls. Red, up-regulation; green, down-regulation; black, no change. G. RT-PCR of pancreas-specific gene expression in mouse livers from GD14 to PND28.

Given that hematopoiesis is carried out in the fetal liver [28], the extent of the transcriptional similarities were determined between the developing liver and other tissues including those involved in hematopoiesis in the adult mouse. We performed an unsupervised comparison by principal components analysis (PCA) between the developing liver and a database of > 80 other mouse tissues which included many involved in hematopoiesis. The fetal and neonatal samples progressed along a trajectory from embryonic stem cells (i.e., Bruce4 and V26 cell lines) to the adult liver (Figure 3A). In contrast to the prediction of similarity to hematopoietic tissues, GD19 samples were more similar to the pancreas from GD18.5 and PND60 mice than to other tissues. Hierarchical clustering of the liver and pancreas samples showed that the fetal liver exhibited greater similarity to the pancreas than neonatal and adult liver (Figure 3B). A direct comparison of the genes altered between the fetal vs. adult livers and the adult pancreas vs. the adult liver demonstrated the impressive overlap in gene expression (Figure 3C). The concordance of the overlapping genes was striking both in direction of change and intensity of the differences (Figure 3D). Pancreas-specific genes (p < 3.16E-05) were identified as detailed in the Materials and Methods and examined for expression changes throughout development (Figure 3E). The genes included many that were up-regulated and not previously associated with the fetal liver. The pancreas-specific genes that were expressed in the fetal liver did not include those that are islet-specific (e.g., islet amyloid polypeptide (*Iapp*), insulin I (*Ins1*), insulin II (*Ins2*), and regenerating islet-derived genes (*Reg1, Reg2, Reg3a, Reg3b*)).

To determine the prevalence of expression of pancreas-related genes in the late term fetus and in the neonate, we examined expression in the livers from male mice at GD19, PND7, PND30 compared to PND67. Many of the up-regulated genes exhibited sustained expression through PND7 including *Reep5, Spink3 *and *Slc39a5 *(Figure 3F). In contrast, many genes encoding digestive enzymes secreted by pancreatic acinar cells were down-regulated in the fetal and neonatal livers including *carboxypeptidase A2, pancreatic (Cpa2), elastase 3, pancreatic (Ela3), carboxypeptidase B1 (tissue) (Cpb1)*, and *colipase, pancreatic (Clps)*.

Expression of four pancreas-specific genes (*Hamp2, Reep5, Slc39a5, Spink3*) was examined by RT-PCR (Figure 3G). *Reep5, Slc39a5 *and *Spink3 *exhibited peak expression between GD14 and GD17 whereas *Hamp2 *was suppressed until PND14. None of these genes appear to have been previously associated with expression in the fetal liver, whereas there is ample evidence for expression in the fetal or adult pancreas. Hepcidin antimicrobial peptide 2 (*Hamp2*) is responsive to dietary iron, indicating a role for *Hamp2 *in the regulation of iron homeostasis [29]. *Hamp2 *expression is sex-dependent, with higher expression in female mouse livers [30], consistent with the higher expression in the maternal liver than in the adult male liver (Figure 3G). Receptor accessory protein 5 (*Reep5*), also known as deleted in polyposis 1 (*Dp1*), is an integral membrane protein that may be involved in shaping the tubular ER [31]. Solute carrier family 39 (metal ion transporter), member 5 (*Slc39a5*) (also known as *Zip5*) belongs to a subfamily of proteins that show structural characteristics of zinc transporters. *Slc39a5 *expression is restricted to many tissues important for zinc homeostasis, including the intestine, pancreas, liver and kidney and localizes to the basolateral surfaces of pancreas acinar and intestinal enterocyte cells in mice fed a zinc-adequate diet. This protein is removed from these cell surfaces and internalized during dietary zinc deficiency, indicating that *Slc39a5 *functions to remove zinc from the blood via the pancreas and intestine, the major sites of zinc excretion in mammals [32]. The serine peptidase inhibitor, Kazal type 3 (*Spink3*), is a trypsin inhibitor, secreted from pancreatic acinar cells into pancreatic juice. *Spink3 *can be detected in the pancreas at GD11.5, before formation of the typical shape of the exocrine structure of the pancreas; acinar cell expression is clearly identified by GD13.5 [33]. Spink3 protein may function to prevent trypsin-catalyzed premature activation of zymogens within the pancreas and the pancreatic duct. Mutations in this gene are associated with hereditary pancreatitis. In Spink3-null mice, the pancreas develops normally up to GD15.5, and starting at GD16.5, there is evidence of autophagic degeneration of acinar cells, but not ductal or islet cells, indicating that *Spink3 *has essential roles in the integrity of pancreatic acinar cells [34]. *Spink3 *is induced in the pancreas after pancreatic injury and its up-regulation may reflect an important endogenous cytoprotective mechanism to prevent further injury [35]. Both *Spink3 *and *Reep5 *are enriched in pancreatic cells over-expressing the pancreatic transcription factor gene *pancreatic-duodenal homeobox 1 *(*Pdx1) *[36]. Additional genes known to be expressed in the pancreas and identified in our study included *Pdia2 *also known as *protein disulfide isomerase (pancreas) like *[37], *phosphoenolpyruvate carboxykinase 2 (mitochondrial) *(*Pck2) *[38] and *phosphoglycerate dehydrogenase *(*Phgdh) *[39].

**Figure 4:**
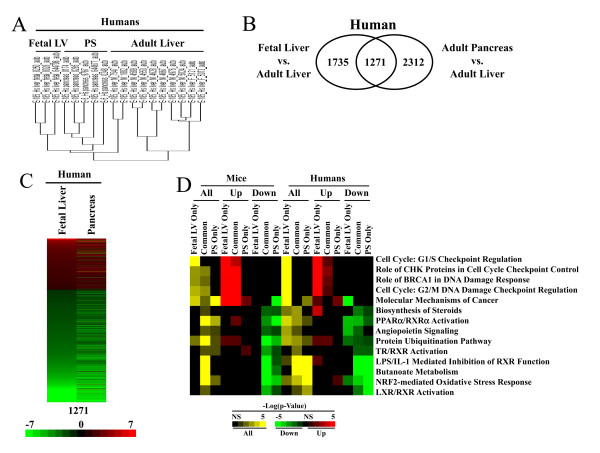
**Transcriptional similarities between the fetal liver and pancreas in humans**. A. Fetal liver exhibits greater similarity to pancreas than adult liver in humans. The biological replicates were clustered using hierarchical clustering. B. Overlap in the genes differentially expressed in the fetal liver or pancreas compared to the adult liver. C. Concordance in the direction and intensity of the fold-changes in the 1271 human overlapping genes from B. The intensity scale indicates fold-changes compared to the adult livers. Red, up-regulation; green, down-regulation. D. Common canonical pathways in mice and humans that are altered in fetal liver and pancreas compared to adult liver. Genes were divided into those depicted in the Venn diagrams in Figure 3C and Figure 4B as 1) expressed in fetal liver only, 2) common to pancreas and fetal liver and 3) in pancreas only. These 3 groups were analyzed using IPA before and after separation into up- and down-regulated genes. The same canonical pathways were compared between mice and humans. The scale is described in Figure 2 legend.

To determine if the human fetal liver also exhibits a pancreas-like signature, a number of human samples were examined from an archived tissue set (Figure 4A). The three fetal liver samples exhibited greater similarity with five adult pancreas samples than with the majority of the adult liver samples. Similar to the mouse results, a direct comparison of the genes which were altered between the fetal vs. adult livers and the adult pancreas vs. the adult liver also showed extensive overlap in humans (Figure 4B), including concordance of the overlapping genes in direction of change and intensity of the differences (Figure 4C).

We next asked whether the pancreas-related genes found in the fetal liver exhibited functional overlap in mice and humans. IPA was used to identify the canonical pathways that were overrepresented by the 3 groups of genes in mice and humans identified in Figures 3C and 4B, respectively. The pathways altered in each species were then compared directly. A number of pathways exhibited similar representation in both mice and humans. The greatest overlap in the up-regulated pathways included those involved in cell proliferation (Cell Cycle: G2/M DNA Damage Checkpoint Regulation, Role of CHK Proteins in Cell Cycle Checkpoint Control, Molecular Mechanisms of Cancer, Role of BRCA1 in DNA Damage Response, and Cell Cycle: G1/S Checkpoint Regulation). These pathways were significantly altered in the fetal liver only, as well as the common genes in both species. In humans and to a lesser extent mice, these pathways were more significant in the fetal liver as expected given the higher level of cell proliferation compared to the adult pancreas. The greatest overlap in the down-regulated pathways included those involved in lipid and steroid homeostasis, and stress responses (Protein Ubiquitination Pathway, NRF2-mediated Oxidative Stress Response, PPARα/RXRα Activation, LPS/IL-1 Mediated Inhibition of RXR Function, Biosynthesis of Steroids, Angiopoietin Signaling, Butanoate Metabolism, LXR/RXR Activation, TR/RXR Activation). These pathways were generally altered in common and pancreas-only gene sets in both species.

These results indicate that the developing liver in mice and humans exhibits transcriptional features similar to the adult pancreas. The fact that genes related to pancreas function are expressed in the neonatal liver is intriguing and prompts the question of whether there is a functional significance to the overlap in the expression of pancreas-specific genes in the neonate. The expression of the pancreas-specific genes in the fetus and neonate does not include those genes encoding pancreatic digestive enzymes from the acinar cells or those associated with the islet cells. Thus, the analysis indicates that the fetal and to a lesser extent the neonatal liver exhibits some transcriptional features of the adult pancreas which may reflect the common embryonic origins of these tissues but not necessarily the inherent functions of the pancreas.

### Identification of a nucleated erythrocyte-specific gene expression signature in the developing liver

**Figure 5:**
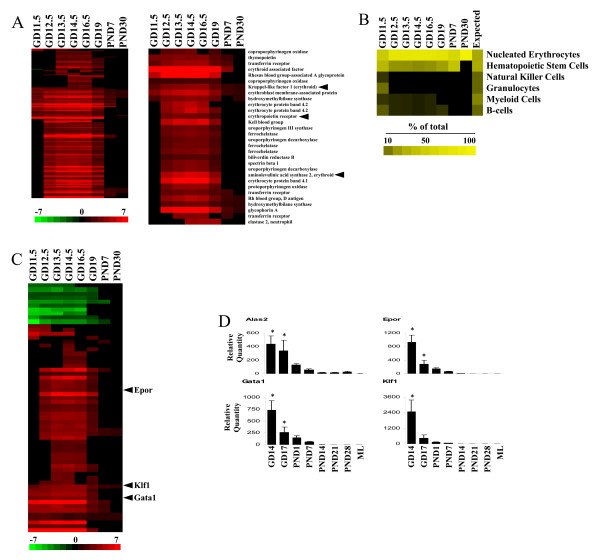
**Identification of a nucleated erythrocyte-specific gene signature in the developing liver**. A. Expression of bone marrow-specific genes in the fetal liver. Left, bone marrow-specific genes identified in the GNF Mouse GeneAtlas V3 dataset were queried for changes in the fetal/neonatal dataset. Right, bone marrow-specific genes expressed in the fetal liver with erythrocyte-associated functions in iron transport and hemoglobin synthesis. Genes confirmed by RT-PCR are indicated. B. Alteration in the signature genes for different blood cell types in the developing liver. The percentage of genes that were altered in each of the groups out of the total number of genes was compared across all of the time points. The values were compared to the expected contribution from each of the cell types based on percentage of total number of genes (right column). The figure shows the greater than expected contribution of the nucleated erythrocyte signature genes to the overall pattern. Changes in the genes for the following cell types or categories were not observed: Differentiated cells, Lymphocytes, Monocytes, Naïve T-cells. The scale represents the percentage of genes from the indicated cell type to the overall gene expression pattern. C. Expression of nucleated erythrocyte-specific genes in the fetal liver. Nucleated erythrocyte-specific genes [16] were examined for expression changes in the fetal/neonatal dataset. Genes confirmed by RT-PCR are indicated. D. RT-PCR confirmation of nucleated erythrocyte-specific gene expression in livers from GD14 to PND28.

The developing liver is a major source of fetal hematopoiesis. A comprehensive identification of liver versus hematopoietic-specific genes during development would be useful to dissect transient or sustained roles for genes in mediating chemical induced effects in the fetal liver. We focused on distinguishing between gene expression changes due to the resident cells of the liver and hematopoietic cells that are transiently present in the fetal liver. Given that many types of blood cells are produced in the bone marrow, adult bone marrow-specific genes (top 500 genes, p < 0.00031) were first identified and then examined for expression changes in the developing liver (Figure 5A, left). These genes uniformly exhibited increased expression compared to adult mice that peaked between GD13.5 and GD16.5. Only a handful of these genes retained elevated expression past GD19, consistent with hematopoietic stem cells migrating from the liver to populate other tissues after birth. Many of the genes possess functions associated with erythrocytes including heme biosynthesis and iron transport (Figure 5A, right).

The individual signatures of specific blood cell types in the fetal liver were examined using marker genes for 10 different blood cell types or categories [19]. Out of the 1418 signature genes for the different blood cell types, a total of 117 genes overlapped with those regulated in the fetal liver. The genes were enriched for nucleated erythrocytes (29 expected but 59 observed) whereas all other cell types except hematopoietic stem cells had less than expected numbers of genes (Figure 5B). Most of the nucleated erythrocyte signature genes exhibited increased expression compared to adult controls (Figure 5C) whereas the hematopoietic stem cell signature genes and genes for other cell types (Additional File 10) were dominated by down-regulated genes.

The expression of four genes (*Alas2, Epor, Gata1, Klf1*) identified as bone-marrow or nucleated erythrocyte-specific were confirmed by RT-PCR. All four genes exhibited similar changes during development that were different from the pancreas-specific genes with peak expression at the earliest measured time (GD14) and decreasing expression until PND14, at which time expression was low or not detectable (Figure 5D). GATA binding protein 1 (*globin transcription factor 1; Gata1*) is a transcription factor that plays an important role in erythroid development by regulating the switch of fetal hemoglobin to adult hemoglobin. The Kruppel-like factor 1 (erythroid) (*Klf1 *or *Eklf1*) encodes a hematopoietic-specific transcription factor that induces high-level expression of adult beta-globin and other erythroid genes. Aminolevulinic acid synthase 2, erythroid (*Alas2*) specifies an erythroid-specific mitochondrially located enzyme. The encoded protein catalyzes the first step in the heme biosynthetic pathway. Drug-induced hemolytic anemia can be detected based on hepatic changes in the expression of genes including *Alas2 *that are mechanistically linked to hematotoxicity [40]. The erythropoietin receptor (*Epor*) is a member of the cytokine receptor family. Upon erythropoietin binding, EpoR activates a kinase-mediated cascade culminating in the activation of erythrocyte-specific transcription factors including Gata1 [41]. A functional *Epor *is likely necessary and sufficient for thrombopoietin to exert its mitogenic effects on erythroid cells [42] and appears to have a role in erythroid cell survival [43]. EpoR with common beta receptor (BetacR) comprise a tissue-protective heteroreceptor that mediates the tissue-protective effects of erythropoietin in preclinical models of ischemic, traumatic, toxic, and inflammatory injuries [44].

The results demonstrate that signature genes for nucleated erythrocytes can be identified within the developing liver and indicate that nucleated erythrocytes exhibit the dominant hematopoietic cell transcriptional signature in the developing liver. Gene and protein expression analysis of a more limited set of tissues during mouse liver development also uncovered features of hematopoiesis in the developing liver [45], and in general their findings are consistent with ours. However, the results from our study indicated that many markers for hematopoiesis were elevated past the latest time point in the Guo et al. study (PND3). Li et al. [13] also examined gene expression at 14 time points across the C57/B6 mouse liver development, and found the gene expression of markers for hematopoiesis peaked from GD12.5 to GD17.5 and then decreased at GD18.5 and older. Our studies provide a foundation on which to examine the effects of different genetic and environmental effects on these genes and their down-stream consequences.

### Impact of development on xenobiotic metabolism gene expression

**Figure 6:**
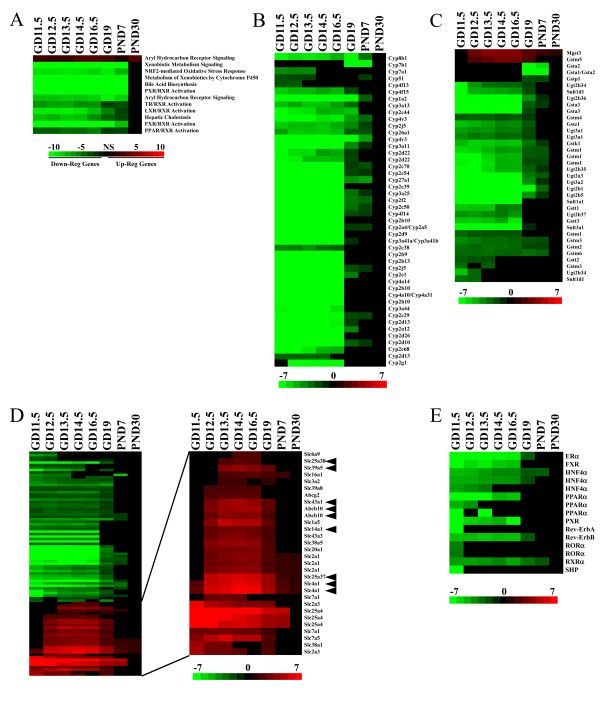
**Impact of liver development on xenobiotic metabolism gene expression**. A.Canonical pathways involved in xenobiotic metabolism. Scale is described in Figure 2. B. Expression of *Cyp *genes. C. Expression of phase II conjugating genes. D. Expression of phase III transporter genes. Right, detail of up-regulated transporter genes. Arrowheads indicate genes identified as being bone marrow-specific and thus may originate from extrahepatic cells. E. Expression of nuclear receptors during development including those that regulate xenobiotic metabolism.

The fetus and neonate are considered potentially sensitive populations to the adverse effects of environmentally relevant chemicals. We were interested, therefore, in characterizing the expression of genes that impact xenobiotic metabolism which may allow follow-on predictions of chemicals to which the fetus or the neonate may be particularly sensitive [46]. We examined canonical pathways involved in xenobiotic metabolism including those controlled by nuclear receptors. Most pathways were down-regulated throughout development (Figure 6A). Aryl hydrocarbon receptor (AhR) signaling was represented by both up- and down-regulated genes, possibly due to the dual role of AhR in liver vascularization during development and xenobiotic metabolism in the adult liver in different cell types [47].

The xenobiotic metabolism genes were separated into phase I cytochrome P450 *Cyp *genes, phase II conjugation enzymes and phase III transporter genes. Remarkably, all *Cyp *genes (Figure 6B) and most phase II genes (Figure 6C) were under expressed relative to the adult animals. Only the phase II genes, *Mgst3 *and *Gstm5*, exhibited increased expression through development. Under expressed genes exhibited discrete times at which they achieved adult expression levels with a few genes achieving adult levels as early as GD14.5 (*Cyp7a1, Cyp4f13, Gstt2, Gstm3, Ugt2b34, Sult1d1*). Most genes achieved adult expression levels after GD16.5. However, even at PND7, the expression of many genes was lower than those in adults.

While genes involved in transport were generally under expressed during development, there were 21 genes which exhibited increased expression. Expression of a number of these phase III genes overlapped with the signature genes for nucleated erythrocytes (*Slc25a10, Slc38a5, Slc43a1, Abcb10, Slc25a38*) [19] or pancreas (*Slc39a5*) (Figure 6D). Transporters with increased fetal expression included genes with known endogenous functions such as transport of amino acids (*Slc1a5, Slc38a1, Slc38a5, Slc3a2, Slc43a1, Slc6a9, Slc7a1, Slc7a5*), adenine nucleotide (*Slc25a4*), glucose (*Slc2a1, Slc2a3*), heme (*Abcb10, Slc25a37, Slc25a38*, all found on the inner mitochondrial membrane), inorganic anion (*Slc4a1(erythrocyte membrane protein band 3, Diego blood group)*), inorganic phosphate (*Slc20a1*), monocarboxylic acids such as lactate (*Slc16a1*), urea (*Slc14a1*), and zinc (*Slc39a5, Slc39a8*). The expression changes for ~40 xenobiotic metabolizing genes in phase I, II and III at GD19-PND67 were confirmed and are published elsewhere [46].

A number of the phase III genes that are coordinately up-regulated during development may have essential roles in liver growth. The amino acid transporters *Slc1a5*, *Slc7a5 *and *Slc3a2 *play roles in regulating the target of rapamycin complex 1 (TORC1), a highly conserved serine/threonine kinase that in mammals activates cell growth in response to stimuli including nutrients (amino acids), growth factors (such as insulin and insulin-like growth factor), and cellular energy status (ATP). Inhibition of TORC1 activates autophagy [48]. The mammalian target of rapamycin (mTOR) canonical pathway was significantly altered during early (p = 2.16E-05) and mid (p = 4.79E-02) expression periods of liver growth (Figure 2A). L-glutamine uptake is regulated by *Slc1a5 *and loss of *Slc1a5 *function inhibits cell growth and activates autophagy. The complex of *Slc7a5/Slc3a2*, acts as a bidirectional transporter that regulates the simultaneous efflux of L-glutamine out of cells and transport of L-leucine/essential amino acids into cells. Thus, L-glutamine flux regulates mTOR to coordinate cell growth and proliferation [49]. In addition to the role of *Slc1a5/Slc7a5/Slc3a2 *in liver growth, other *Slc *family members that were up-regulated during development may also have roles. Immunomodulatory compounds that inhibit human and rat T lymphocyte proliferation act by inhibiting *Slc16a1 *[50]. The essential role of *Slc20a1 *in liver development was determined in *Slc20a1*-null mice which displayed decreased proliferation, extensive apoptosis in the liver and embryonic lethality at GD12.5 [51], the time of earliest expression changes observed by microarray (Figure 6D, right).

To begin to address the basis for the low level of expression observed for many xenobiotic metabolism genes, the expression of transcription factors that are known to mediate chemical-inducible gene expression was examined. Many of the nuclear receptors are targets of chemicals and drugs. A subset of nuclear receptors exhibited decreased expression compared to PND67 animals (Figure 6E). The expression of components of the dioxin receptor (*Ahr*, *Arnt *family members) and *Nfe2l2*, also known as *Nrf2*, (*Keap1, Maf *family members) did not exhibit changes throughout development. We hypothesize that the expression of xenobiotic metabolism genes is reduced relative to the adult due to lower expression of the factors that control their basal transcription levels. One such factor could be the nuclear receptor *Hnf4a *which controls the expression of a large number of liver-specific genes [52]. Like our microarray results, Li et al. [13] observed an increase in the expression profile of *Hnf4a *during mouse liver development, with enhanced expression occurring at postnatal stages.

Our comprehensive analysis of XME expression adds to the current body of knowledge which indicates that development affects the hepatic expression of XMEs in mice and rats. A comparative expression profiling of 40 mouse cytochrome P450 genes in GD7, GD11, GD15, and GD17 Swiss Weber/NIH embryos was conducted using multiple tissue Clontech cDNA panel Mouse I [53]. Twenty-seven P450s were expressed during development with numbers gradually increasing throughout development. *Cyp2s1*, *Cyp8a1*, *Cyp20*, *Cyp21a1*, *Cyp26a1*, *Cyp46*, and *Cyp51 *were detected at all stages. In rats, there is a 4- and 6- fold increase in CYP content at postnatal days 7 and 14, respectively, compared with day 1 of birth [54]. CYP1A1 is expressed during early gestation, but expression of most of the other CYP enzymes occurs at or near birth (CYP2B, CYP2C23, CYP3A) or immediately after birth (CYP2E1) [55]. CYP1A2, CYP2C6, CYP2C11, CYP2C12, and CYP4A10 are expressed after the first week of birth [55,57,54,56]. CYP2B1 activity at PND4 is comparable to levels observed in adult livers [54], whereas postnatal [55] activity of CYP2E1 increases linearly with age and at PND30 is comparable to that in adult liver [55,54]. In humans, total cytochrome P450 content in the fetal liver is between 30% and 60% of that found in the adult and approaches adult values by 10 years of age [58]. CYP3A7 activity is high immediately after birth; during the first days after birth there is a shift from mainly CYP3A7 activity to CYP3A4 activity [59]. CYP2D6 and CYP2E1 activities are minimal in the fetal liver but quickly increase hours after birth [60,61]. Sonnier et al. reported delayed ontogenesis of CYP1A2 in the human liver [62].

In summary, analysis of xenobiotic metabolism genes indicates that there are profound differences in their expression compared to the young adult. While there are a number of genes that exhibit increased expression compared to the adult, the vast majority of genes exhibit decreased levels. These decreases could potentially result in prolonged chemical effects including toxicity in the fetus due to inability to metabolize and excrete xenobiotics. Predictions of chemical sensitivities can be made by identifying chemicals that interact with individual XMEs. This would include XMEs involved in the metabolism of a chemical whose expression is decreased during early life stages. However, depending on the chemical, there may be cases where decreased expression of a CYP may be protective of the fetus. For example, CYP1A2 metabolically activates aflatoxin B1 to its carcinogenic metabolite [63]. Future work will be directed towards determining the chemicals to which the fetus and the neonate may exhibit altered responses and will depend in part on accounting for the effects of tissues that act as a metabolic barrier to environmental exposure to protect the embryo (yolk sac) and the fetus (placenta).

## Conclusions

Our microarray analysis demonstrates that the fetal liver undergoes dramatic transcriptional changes during development and maturation. We identified key pathways that support the growth and function of the developing liver. Signature genes for erythropoiesis were identified and indicate that nucleated erythrocytes exhibit the dominant hematopoietic cell transcriptional signature in the developing liver. Our results also indicate that the developing liver exhibits transcriptional features similar to the adult pancreas, which may reflect the common embryonic origins of these tissues. The vast majority of genes associated with xenobiotic metabolism and transport exhibit decreased expression compared to the adult which may alter susceptibility to environmental chemicals in the fetus and neonate. Our analysis also emphasizes that microarray datasets can be successfully combined to create a broader view of gene expression changes during development.

## List of abbreviations

Ct: cycle threshold; PCR: polymerase chain reaction; XME: xenobiotic metabolizing enzyme.

## Authors' contributions

WW, BV, and HR analyzed the microarray data, GK generated and analyzed the RT-PCR data. BA and SK performed animal experiments. SK and KH generated microarray and in situ hybridization data. JL and JCC conceived of the study, participated in study design and animal studies, analyzed microarray data and helped to draft the manuscript. All authors read and approved the final manuscript.

## Supplementary Material

Additional file 1 **Source bias removal demonstrated with RNA spike-in controls**. 64 Affymetrix hybridization control probesets were analyzed with principal component analysis before and after the application of Distance Weighted Discrimination. Red, GD19-PND67 samples from the EPA dataset. Black, GD11.5-16.5 samples from the Otu et al. [13] dataset. Click here for file

Additional file 2 **Expression of actin and GAPDH during mouse liver development**. RT-PCR of gene expression in livers from GD14 to PND28. Click here for file

Additional file 3 **Expression changes in fetal liver genes**. Genes which exhibited significant differences (p < 2.55E-08) between fetal liver and all adult tissues are shown. Click here for file

Additional file 4 **Expression of *Makorin 1 *and *Regulator of Differentiation 1 *by microarray and in situ hybridization**. Figure S1: Expression of the probesets for *Mkrn1 *and *Rod1 *through development. Figures S2-S6: In situ hybridization of *Mkrn1 *or *Rod1 *at the indicated times during development. L, liver; Lu, lung; St, stomach; M, metanephros; SC, spinal cord; DRG, dorsal root ganglion; LA, atrium; NE, neuroepithelium of neural tube; MT, mesonephric tubules; UB is ureteric bud. Click here for file

Additional file 5 **Gene expression changes in the livers of fetal and neonatal mice**. Gene expression changes during development of the mouse liver (GD11.5, GD12.5, GD13.5, GD16.5, GD19, PND7, PND30). Click here for file

Additional file 6 **Gene expression changes during discrete windows of development in the livers of fetal and neonatal mice**. Classification of genes altered during mouse liver development (early, mid, late, sustained). Click here for file

Additional file 7 **Canonical pathways involved in signaling altered during liver development**. Canonical pathway information was extracted from Ingenuity. The scale numbers are the -log(p-value) and range from < 10^-7 ^to not significant (NS). Red, up-regulated pathways; green, down-regulated pathways; black, no change. Click here for file

Additional file 8 **Pathways involved in liver toxicity are altered during liver development**. Pathways mentioned in the text are indicated by arrowheads. The scale numbers are the -log(p-value) and range from < 10^-7 ^to not significant (NS). Red, up-regulated pathways; green, down-regulated pathways; black, no change. Click here for file

Additional file 9 **Alterations in purine and pyrimidine metabolism genes at GD16.5**. Figure S7: The KEGG metabolic map of purine metabolism was extracted from IPA. Figure S8: The KEGG metabolic map of pyrimidine metabolism was extracted from IPA. Pink diamonds indicate the genes that exhibited increased abundance. Click here for file

Additional file 10 **Expression of marker genes for hematopoietic cell types**. Marker genes from Chambers et al. [16] were used to query expression across development for hematopoietic stem cells (HSC) (Left) and all genes from cell types other than HSC and nucleated erythrocytes that exhibited changes during development (Right). Click here for file
