# Supplementary figures and images for: Transcriptional ontogeny of the developing liver
Source: BMC Genomics. 2012 Jan 19;13:33. doi: 10.1186/1471-2164-13-33 (PMC3306746; doi:10.1186/1471-2164-13-33)

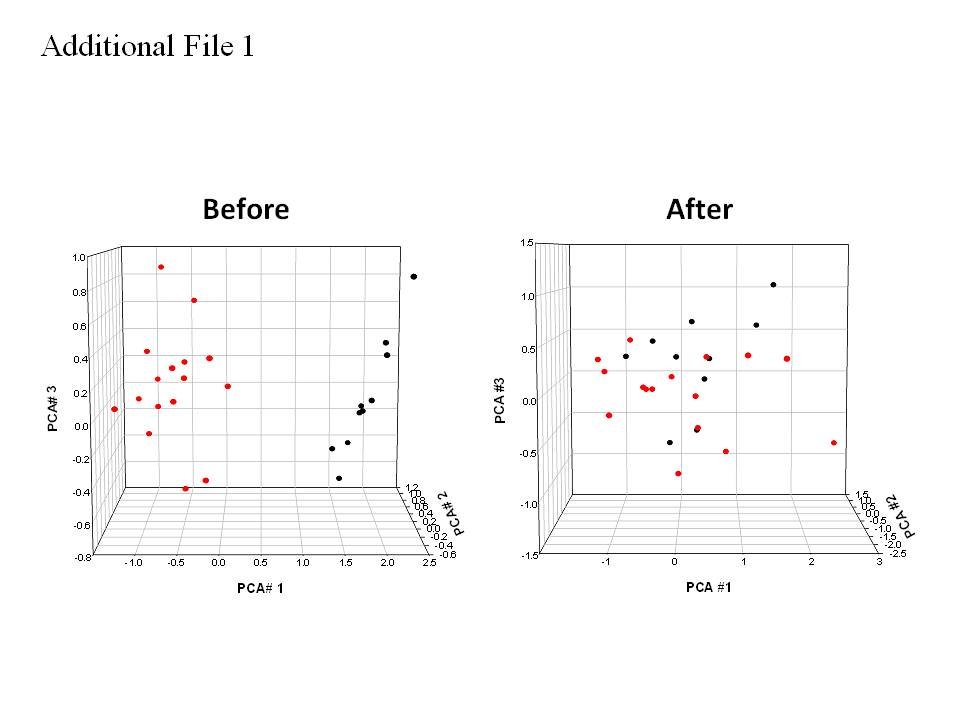

Supplement: Additional file 1 — Source bias removal demonstrated with RNA spike-in controls. 64 Affymetrix hybridization control probesets were analyzed with principal component analysis before and after the application of Distance Weighted Discrimination. Red, GD19-PND67 samples from the EPA dataset. Black, GD11.5-16.5 samples from the Otu et al. [13] dataset. [file 1471-2164-13-33-S1.JPEG]

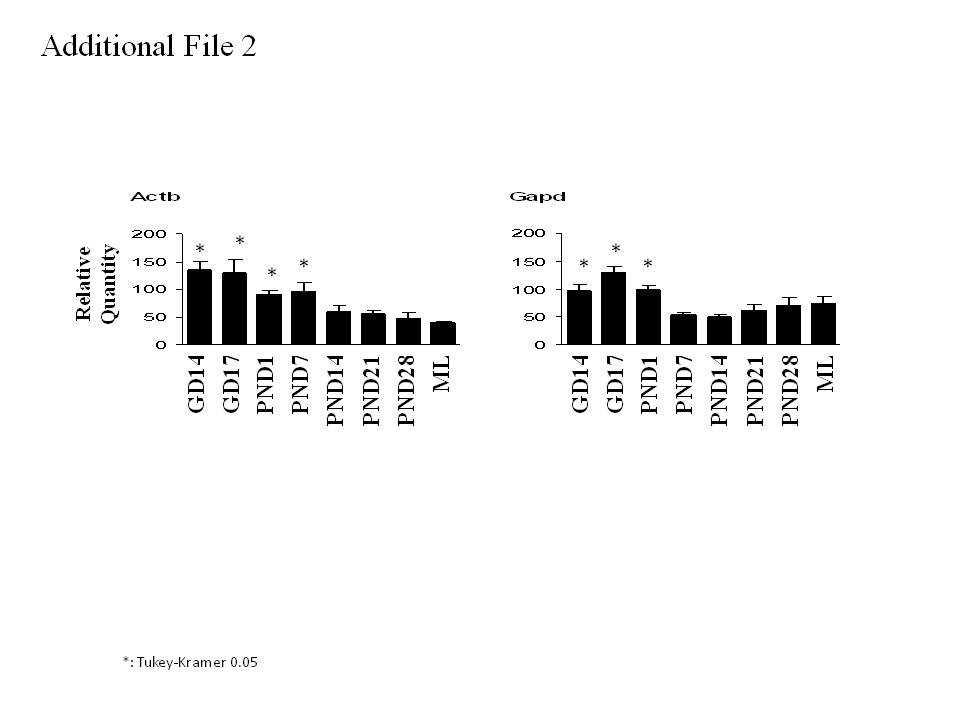

Supplement: Additional file 2 — Expression of actin and GAPDH during mouse liver development. RT-PCR of gene expression in livers from GD14 to PND28. [file 1471-2164-13-33-S2.JPEG]

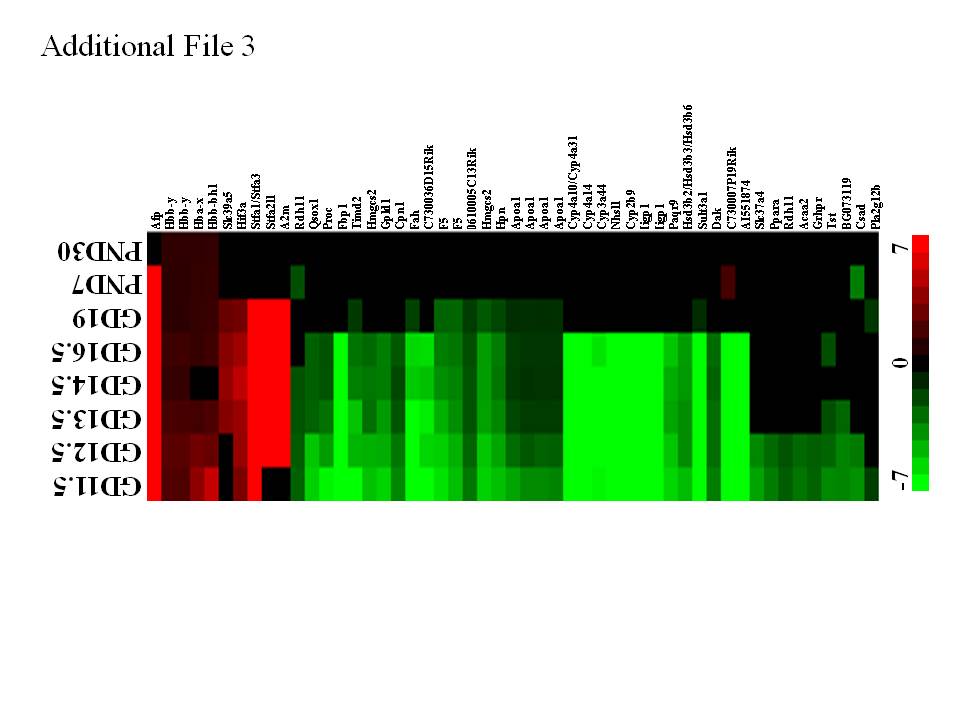

Supplement: Additional file 3 — Expression changes in fetal liver genes. Genes which exhibited significant differences (p < 2.55E-08) between fetal liver and all adult tissues are shown. [file 1471-2164-13-33-S3.JPEG]

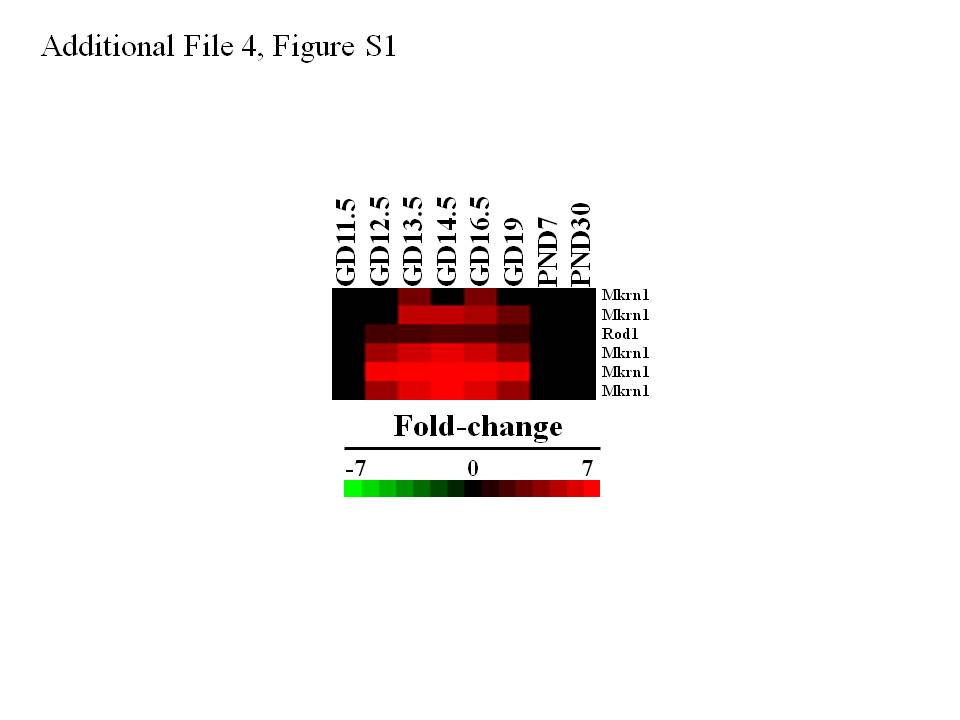

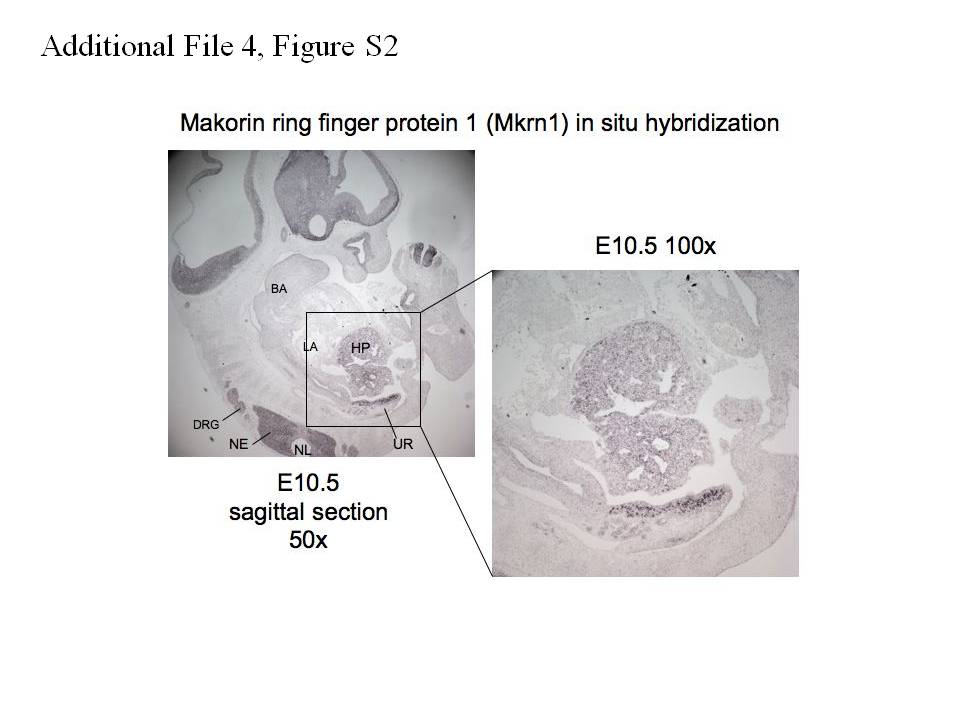

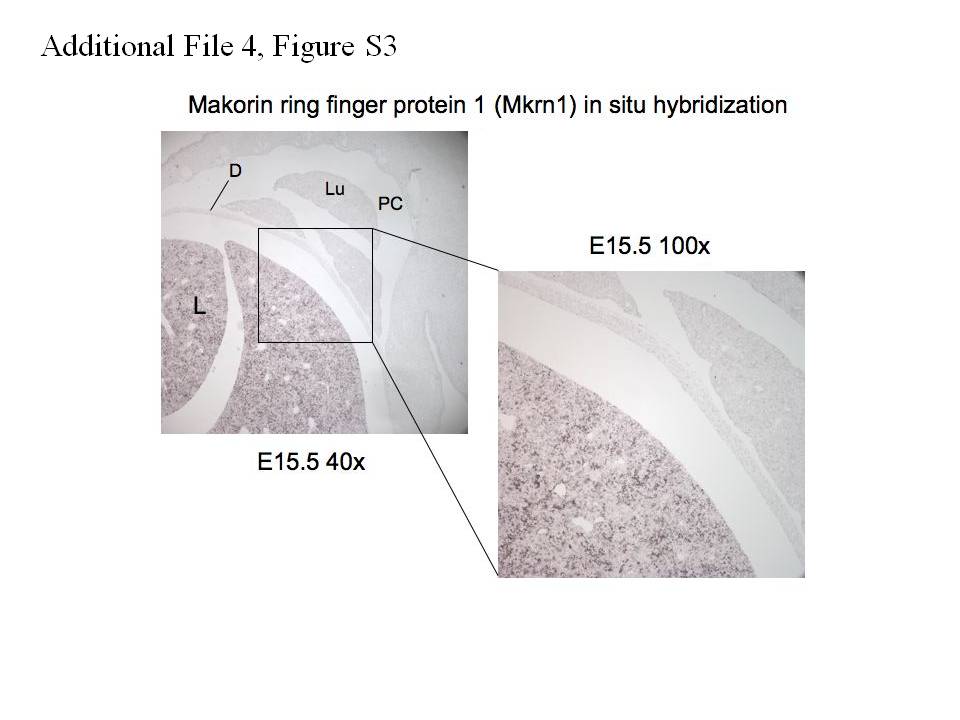

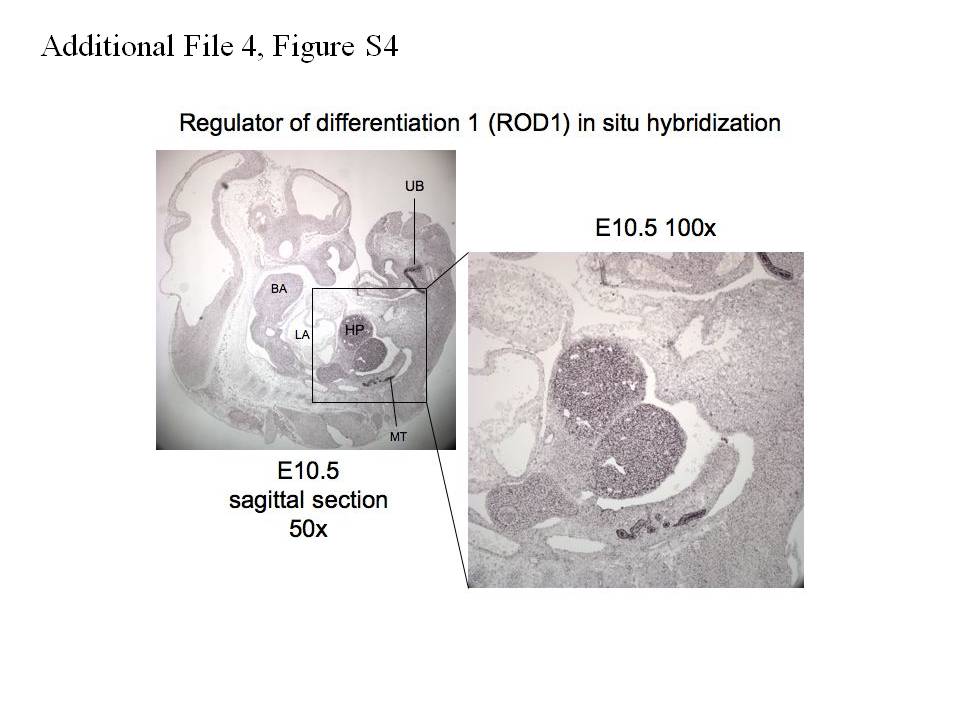

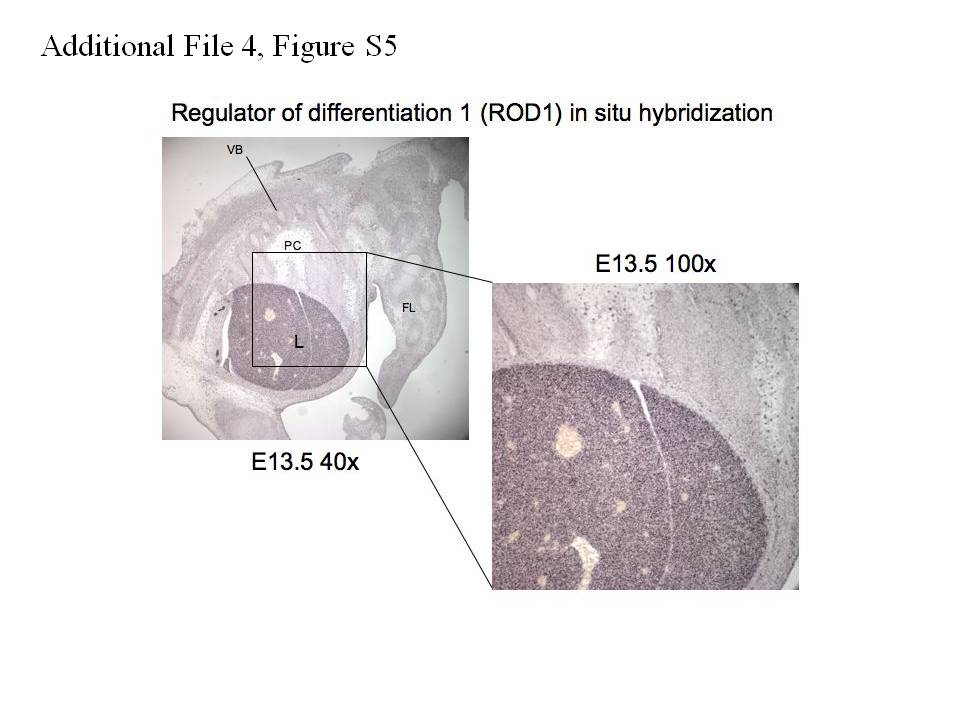

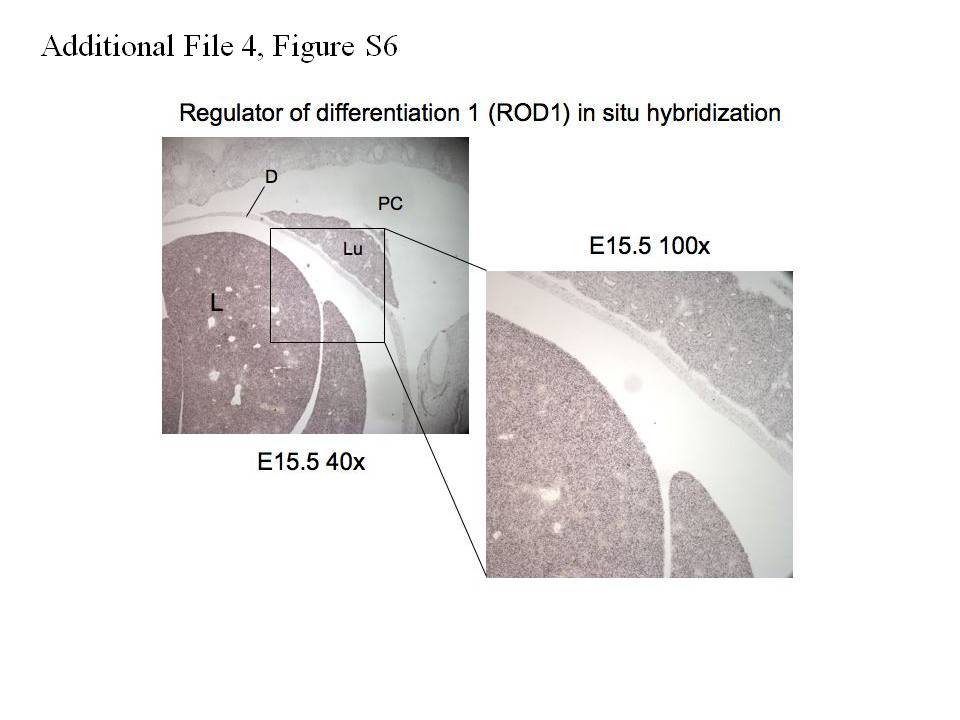

Supplement: Additional file 4 — Expression of Makorin 1 and Regulator of Differentiation 1 by microarray and in situ hybridization. Figure S1: Expression of the probesets for Mkrn1 and Rod1 through development. Figures S2-S6: In situ hybridization of Mkrn1 or Rod1 at the indicated times during development. L, liver; Lu, lung; St, stomach; M, metanephros; SC, spinal cord; DRG, dorsal root ganglion; LA, atrium; NE, neuroepithelium of neural tube; MT, mesonephric tubules; UB is ureteric bud. [file 1471-2164-13-33-S4.DOC]

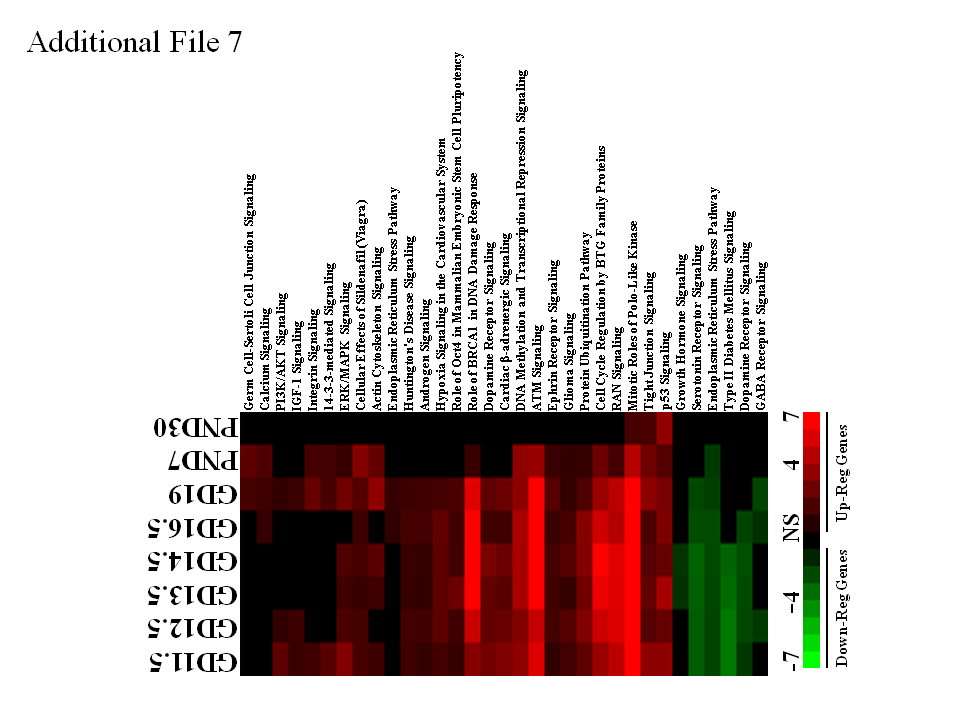

Supplement: Additional file 7 — Canonical pathways involved in signaling altered during liver development. Canonical pathway information was extracted from Ingenuity. The scale numbers are the -log(p-value) and range from < 10-7 to not significant (NS). Red, up-regulated pathways; green, down-regulated pathways; black, no change. [file 1471-2164-13-33-S7.TIFF]

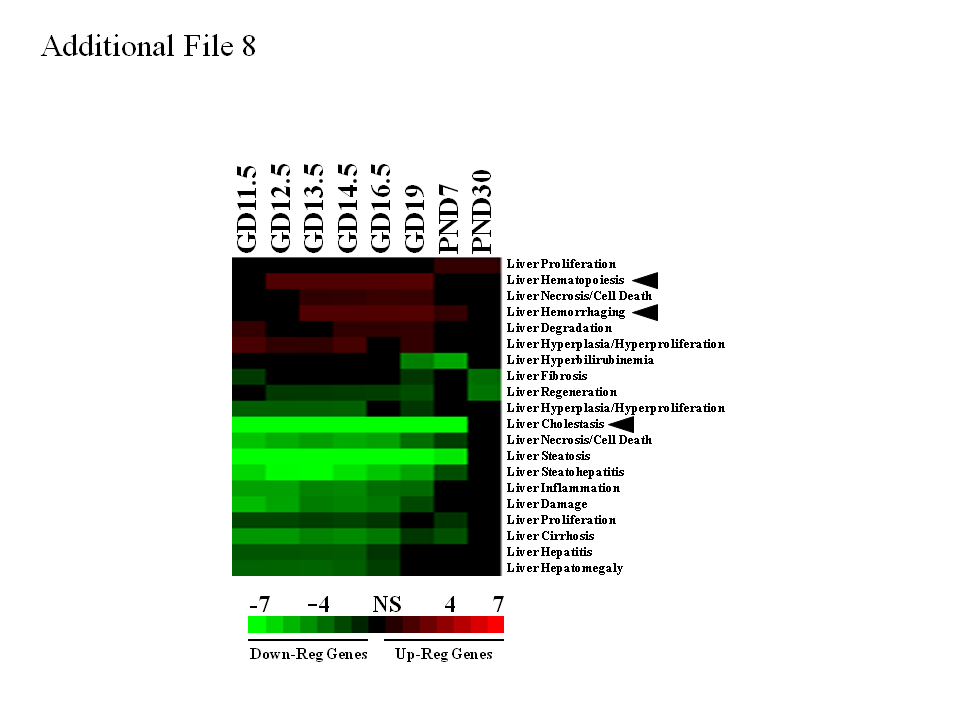

Supplement: Additional file 8 — Pathways involved in liver toxicity are altered during liver development. Pathways mentioned in the text are indicated by arrowheads. The scale numbers are the -log(p-value) and range from < 10-7 to not significant (NS). Red, up-regulated pathways; green, down-regulated pathways; black, no change. [file 1471-2164-13-33-S8.TIFF]

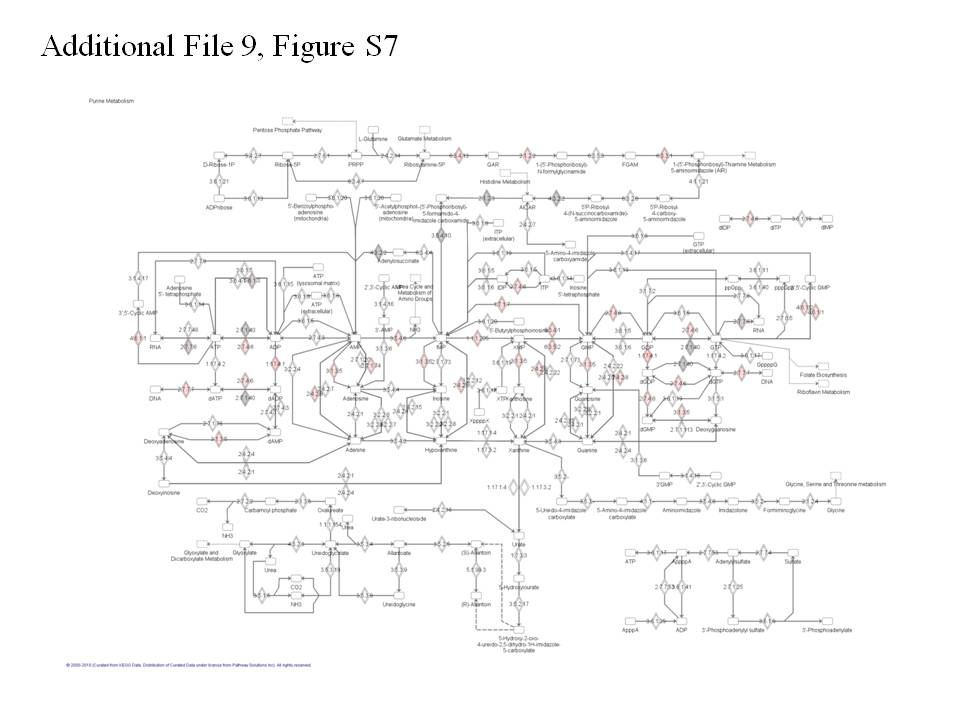

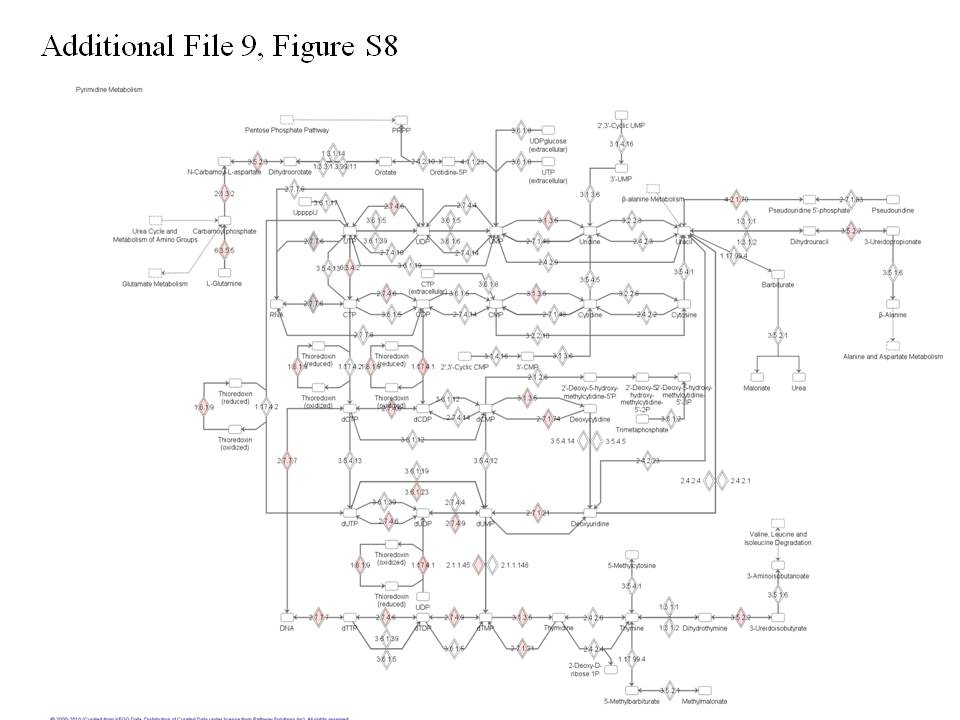

Supplement: Additional file 9 — Alterations in purine and pyrimidine metabolism genes at GD16.5. Figure S7: The KEGG metabolic map of purine metabolism was extracted from IPA. Figure S8: The KEGG metabolic map of pyrimidine metabolism was extracted from IPA. Pink diamonds indicate the genes that exhibited increased abundance. [file 1471-2164-13-33-S9.DOC]

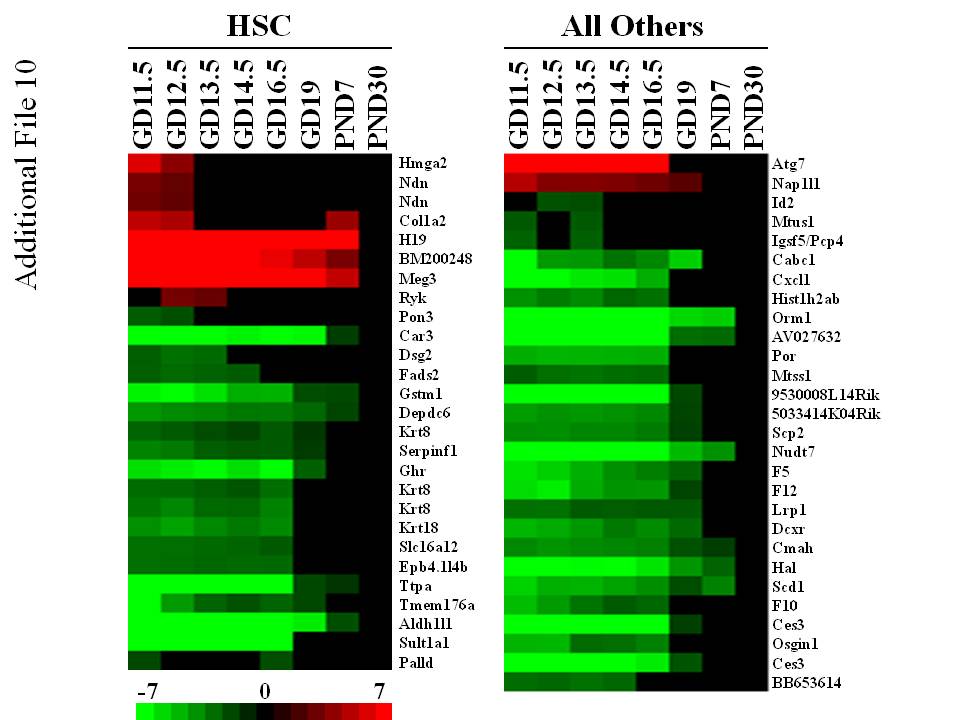

Supplement: Additional file 10 — Expression of marker genes for hematopoietic cell types. Marker genes from Chambers et al. [16] were used to query expression across development for hematopoietic stem cells (HSC) (Left) and all genes from cell types other than HSC and nucleated erythrocytes that exhibited changes during development (Right). [file 1471-2164-13-33-S10.JPEG]
